# Effect of estradiol on enzymes of vascular extracellular nucleotide metabolism

**DOI:** 10.1007/s42000-020-00242-6

**Published:** 2020-09-15

**Authors:** Areta Hebanowska, Paulina Mierzejewska, Alicja Braczko

**Affiliations:** Department of Biochemistry, Medical University of Gdansk, Gdansk, Poland

**Keywords:** Estrogens, CD39, CD73, eADA, Endothelium, Aorta, Mice

## Abstract

**Purpose:**

Estrogens have beneficial effects on the cardiovascular system, promoting vasodilation, endothelial cells growth, relaxation, and regulation of blood pressure. Some of these effects could be associated with the purinergic system known for the control of vasodilation, inflammation, and platelet function. The aim of our study was the evaluation of ATP, AMP, and adenosine extracellular catabolism, catalyzed by ectonucleoside triphosphate diphosphohydrolase-1 (CD39), ecto-5′-nucleotidase (CD73), and ecto-adenosine deaminase (eADA) in mouse aortas.

**Methods:**

Extracellular hydrolysis of ATP, AMP, and adenosine was estimated on the aortic surface of 3-month-old female and male C57BL/6 J wild-type (WT) mice, in female WT mouse aortas incubated for 48 h in the presence or absence of 100 nM estradiol, and in WT female mouse and ApoE-/-LDL-R-/- aortas. The conversion of substrates to products was analyzed by high-pressure liquid chromatography (HPLC).

**Results:**

We demonstrated significantly higher adenosine deamination rate in WT male vs. female mice (*p* = 0.041). We also noted the lower adenosine hydrolysis in aortas exposed to estradiol, as compared with the samples incubated in estradiol-free medium (*p* = 0.043). Finally, we observed that adenosine conversion to inosine was significantly higher on the surface of ApoE-/-LDL-R-/- aortas compared with WT mice (*p* = 0.001). No such effects were noted in ATP and AMP extracellular hydrolysis.

**Conclusion:**

We conclude that estradiol inhibits the extracellular degradation of adenosine to inosine, which may be an element of its vascular protective effect, as it will lead to an increase in extracellular adenosine concentration. We can also assume that during the development of the atherosclerotic process, the protective role of estradiol in the regulation of adenosine degradation may be obscured by other pathogenic factors.

## Introduction

The extracellular nucleotides, ATP and ADP, and the nucleoside, adenosine, have been shown to be regulators of vascular function. They are released from various cells, including endothelial cells (ECs), vascular smooth muscle cells (VSMCs), platelets, and immune cells [1]. They control the vascular tone and modulate the inflammatory processes, acting via cell surface receptors [2]. ATP and ADP, which operate through the P2 family of receptors, are considered vasoconstriction, proatherosclerotic, and proinflammatory response mediators. Adenosine (and putatively AMP) counteracts those effects through P1 receptors [3]. Extracellular concentration of adenine nucleotides and adenosine is modulated by ectoenzymes found in plasma and on the cell surface [4, 5]. ATP released from cardiovascular system (CVS) cells is sequentially degraded to ADP and, finally, to AMP mainly by CD39 (ectonucleoside triphosphate diphosphohydrolase-1; Entpd1) [6, 7]. AMP as a product of CD39 action is then metabolized by CD73 (5′-ectonucleotidase; NT5E), which produces adenosine [5, 8]. Adenosine is taken up by the cells through specific transporters (ENT) and later reused for intracellular nucleotide resynthesis or degraded extracellularly to inosine by adenosine deaminase (ADA) [9, 10]. Degradation and cellular reuptake of adenosine limit the duration of adenosine P1 receptor activation [11]. There are additional enzymes involved in extracellular purine nucleotides and adenosine metabolism. Ectonucleoside pyrophosphatase/phosphodiesterase 1 (ENPP1) is an enzyme of a wide range of substrates and can degrade ATP directly to AMP, though it plays a minor function in the purinergic signaling system [12]. Adenosine, which is mainly a product of CD73 or alkaline phosphatase, is also supplied by the pathway of cAMP degradation through the combined action of ecto-phosphodiesterase or alkaline phosphatase and CD73 [2]. Regulation of these enzymes’ activities is important for the proper control of vascular homeostasis. Increased concentrations of ATP and ADP, resulting from the inhibited activity of CD39, and decreased concentration of adenosine, resulting from the inhibited activity of CD73, are observed in different vascular pathologies, including atherosclerosis, aortic stenosis, inflammation, pathological angiogenesis, hypoxia, and ischemia [13–16]. Increased activity of ADA leading to the diminished concentration of adenosine is observed in atherosclerosis and hypoxia [10, 17].

Some earlier investigations showed that estrogens might be involved in the control of the extracellular purinergic system. Estrogen is well-known for its protective effects on the cardiovascular system, though the underlying mechanism of these effects remains elusive [18, 19]. The prevalence of cardiovascular diseases among men is much greater compared with that in premenopausal females, though after menopause the risk for atherosclerosis and similar diseases increases significantly in females. It is already established that estrogens influence the function of the cells of the cardiovascular system, including platelets, leukocytes, vascular muscle, and endothelial cells. They induce vasodilation, induce the migration of endothelial cells, and decrease the inflammatory response and platelet aggregation [20, 21]. There is some evidence of the possible involvement of estrogens in the regulation of purine extracellular metabolism. Deprivation of estrogens by ovariectomy leads to increased hydrolysis of ATP, ADP, and AMP in rat blood serum, while estrogen replacement therapy exerts the opposite effect [22]. Ovariectomy and subsequent decrease of estrogen levels lead to decreased catabolism of ATP and ADP by CD39 and activity of CD73, both attached to the platelet plasma membrane [23]. The influence of estradiol on extracellular purine metabolism was also shown in hippocampal synaptosomes [24, 25] and breast cancer cells [26]. So far, limited data are available on the effects of estrogen on vascular extracellular purine metabolism. Xu et al. have demonstrated that pial arteriolar vasodilation promoted by ADP remains under the control of estrogen in female rats [27].

Up till now, there is no data on the impact of estrogens on the purinergic system of the aorta; thus, our study aimed to explore this field. We investigated the hydrolysis of purine nucleotides and adenosine deamination in intact aortas isolated from female and male mice. We also analyzed the influence of estradiol on purine nucleotides and adenosine metabolism in the intact aorta of female mice. Finally, because both estrogens and purine compounds are involved in the development of cardiovascular pathology, we estimated hydrolysis of ATP/ADP, AMP, and adenosine in atherosclerotic female mice with knockout of LDL receptor and ApoE genes.

## Methods

### Animals and housing

Female and male mice (aged 3 months) C57BL/6 J wild-type (WT) and female double knockouts for apolipoprotein E (ApoE) and low-density lipoprotein receptor (LDL-R) on the C57BL/6 J background (ApoE-/-LDL-R-/-) were bred in house and used for the experiments. The mice were housed in a 12/12 h light/dark cycle in environmentally controlled rooms (23 ± 1 °C, 40 ± 5% humidity). The animals had unlimited access to water and a standard chow diet.

The mice were sacrificed under anesthesia by a ketamine-xylazine (100 mg/kg) intraperitoneal injection. Isolated fragments of the aorta (one aorta fragment from each animal) were cleaved from perivascular adventitia in cold HBSS, cut along to expose the inner surface, and used for measurements of the extracellular nucleotides and adenosine conversion rates.

### Extracellular nucleotide and adenosine breakdown analyses

Analyses of extracellular nucleotides and adenosine metabolism were done in intact aortas isolated from WT female mice, treated with estradiol, from WT male and female mice, and ApoE -/- LDL-R -/- female mice.

Immediately after the dissection, fragments of WT female thoracic aorta were immersed in 1 ml of Hanks Balanced Salt Solution (HBSS Sigma Aldrich, Poznan) phenol-red free and opened longitudinally by an incision along its ventral aspect. The aortic fragments were immersed in 1 ml of HBSS phenol-red free containing 100 nM estradiol (*n* = 6) for 48 h. Control samples (*n* = 6) were placed in HBSS only. After 48 h of incubation, the aortic sections were washed and placed in 24-well plates with 1 ml of HBSS phenol-red free and preincubated for 15 min. After preincubation, substrates suitable for each extracellular enzyme were sequentially added: 50 μM of adenosine for adenosine deaminase (eADA), 50 μM of ATP for ectonucleoside triphosphate diphosphohydrolase-1 (eNTPD, CD39), and 50 μM of AMP for 5′-ectonucleotidase (NT5E) (ATP, ADP, and adenosine—Sigma Aldrich, Poznan, Poland). The aorta sections were washed twice, each time with 1 ml of HBSS, after each incubation with a particular substrate. After 0, 5, 15, and 30 min of incubation at 37 °C, samples (50 μl) were collected and then directly analyzed using high-performance liquid chromatography (HPLC). Analysis of ATP and AMP hydrolysis was conducted in the presence of adenosine deaminase inhibitor (erythro-9-(2-hydroxy-3-nonyl) adenine; EHNA—Sigma Aldrich, Poznan, Poland) at a concentration of 5 μM.

The concentration of nucleotides and nucleosides was measured by reversed-phase HPLC [28]. The rates of ATP and AMP hydrolysis, as well as adenosine deamination, were calculated from a linear phase of the reaction and normalized for the mass of aorta, which was measured immediately after the incubation with ectoenzyme substrates.

Immediately after the dissection, purified aorta fragments of WT female and male mice and aortas of female ApoE-/-LDL-R-/- mice were immersed in 1 ml of HBSS, containing substrates for ectoenzymes, for measurements of enzyme activities, as described earlier.

All experiments were conducted following the Guide for the Care and Use of Laboratory Animals published by the European Parliament, Directive 2010/63/EU, and were approved by the Local Bioethical Committee.

### Statistical analysis

Values are presented as median and range. Statistical analysis was performed using the nonparametric Mann-Whitney *U* test to assess differences between the two groups, and a *p value < 0.05 was considered a significant difference.

## Results

### Extracellular ATP, AMP, and adenosine catabolism in aortas of WT female and male mice

**Fig. 1:**
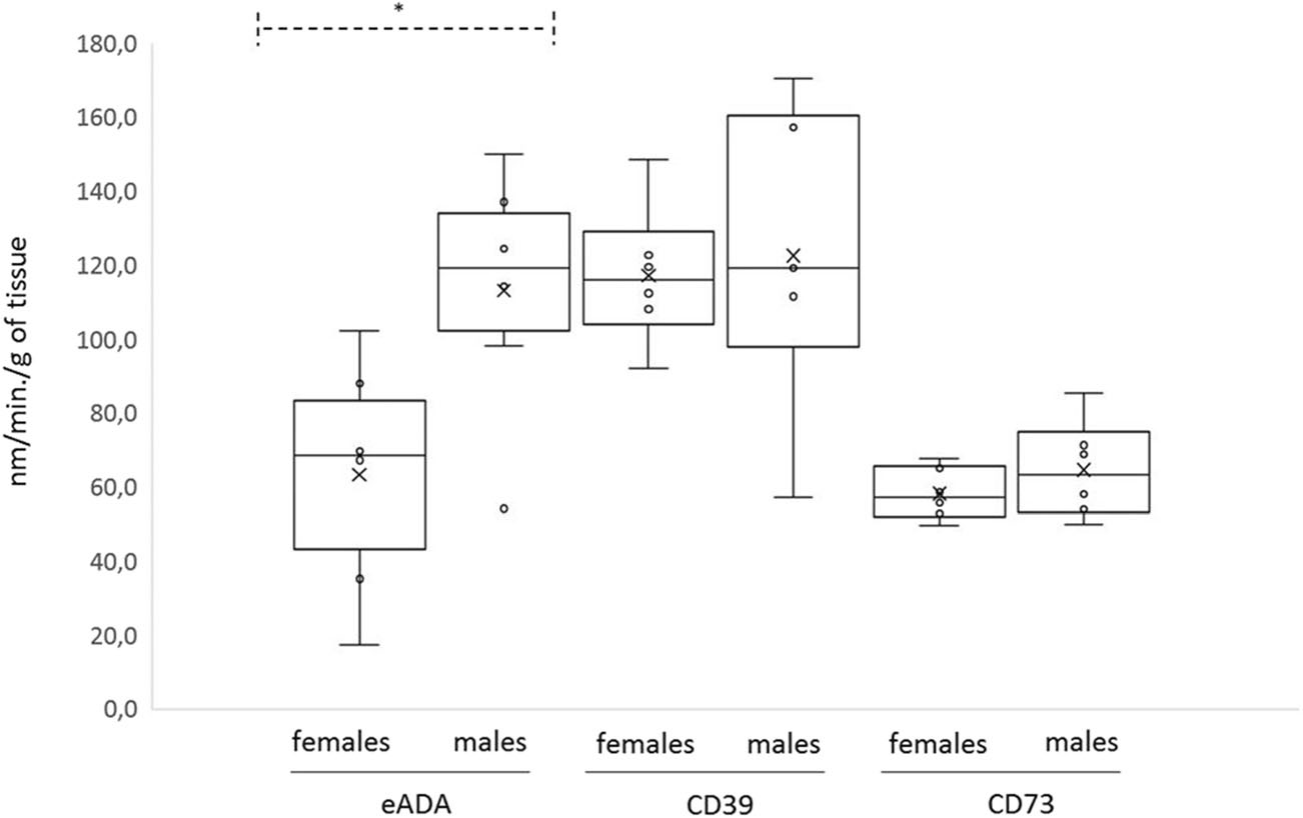
Comparison of eADA, CD73, and CD39 activities in WT female (F) (*n* = 6) and male (M) (n = 6) mice aortic endothelial cells. Data are presented as a boxplots

We measured activities of three extracellular enzymes involved in the catabolism of adenine nucleotides and adenosine in aortas of WT male (M) and female (F) mice (*n* = 6 in both groups). The results showed no differences between sexes as regards CD39 and CD73 activity (Table 1, Fig. 1). In contrast, the activity of ADA was twice as high in males, 119.2 (98.1–150.1) nm/min/g of tissue, median (range, min.-max.), as in females, 68.2 (17.2–102.3) nm/min/g of tissue, *p* = 0.041, which may result in a higher velocity of adenosine degradation in male aortas (Table 1, Fig. 1).

**Table 1:** Activities of aortal endothelium ectoenzymes (eADA, CD39, and CD73) in mice

Ectoenzyme	WT females	WT males	*p* value
eADA	68.38 (17.2–102.3)	119.2 (98.1–150.1)	0.041*
CD39	115.3 (91.91–148.6)	119.1 (57.27–170.3)	0.686
CD73	57.2 (49.53–67.55)	63.32 (49.91–85.45)	0.423
	WT females/E-	WT females/E+	*p* value
eADA	50.91 (22.15–78.45)	22.31 (14.21–27.95)	0.043*
CD39	128.9 (89.27–210.5)	144.7 (58.25–254.0)	0.589
CD73	55.11 (41.01–101.6)	70.93 (55.13–84.56)	0.18
	WT females	Females LDL-R-/-ApoE-/	*p* value
eADA	36.6 (11.21–145.7)	161.3 (79.1–257.7)	0.001*
CD39	117.5 (91.9–148.5)	134.6 (58.25–210.6)	0.247
CD73	53.71 (32.71–99.84)	46.72 (32.71–57.55)	0.301

Data are presented as median (range). A nonparametric test was used (Mann-Whitney *U* test). A *p* value of <  0.05 was considered significant (*). WT, wild type; Females/E+, female mice aortas treated with estradiol; Females/E-, female mice aortas untreated with estradiol; Females ApoE-/-LDL-R-/-, female mice with genetic deficiency of LDL receptor and ApoE

### Influence of estradiol on extracellular ATP, AMP, and adenosine catabolism in WT female aortas

**Fig. 2:**
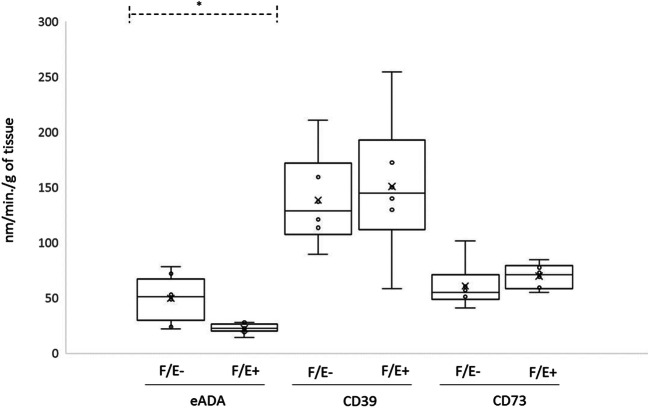
Activities of ADA, CD73, and CD39 in WT female mice aortic endothelial cells after treatment with 100 nM estradiol (F/E+) (*n* = 6) vs. controls (F/E-) (*n* = 6). Data are presented as a boxplots

After 48 h of incubation of female aortas (*n* = 6 in both groups) in medium containing 100 nM estradiol (F/E+) or in medium with no estradiol (F/E-), the results showed no differences in CD39 and CD73 activities (Table 1, Fig. 2). eADA activity was markedly lower in the presence of estradiol—22.31 (14.21–27.95) nm/min/g of tissue in F/E+ vs. 50.91 (22.15–78.45) nm/min/g of tissue in F/E-, *p* = 0.043 (Table 1, Fig. 2).

### Extracellular ATP, AMP, and adenosine catabolism in WT aortas (F control) and female ApoE-/-LDL-R-/- (F ApoE-/-LDL-R-/) mice

**Fig. 3:**
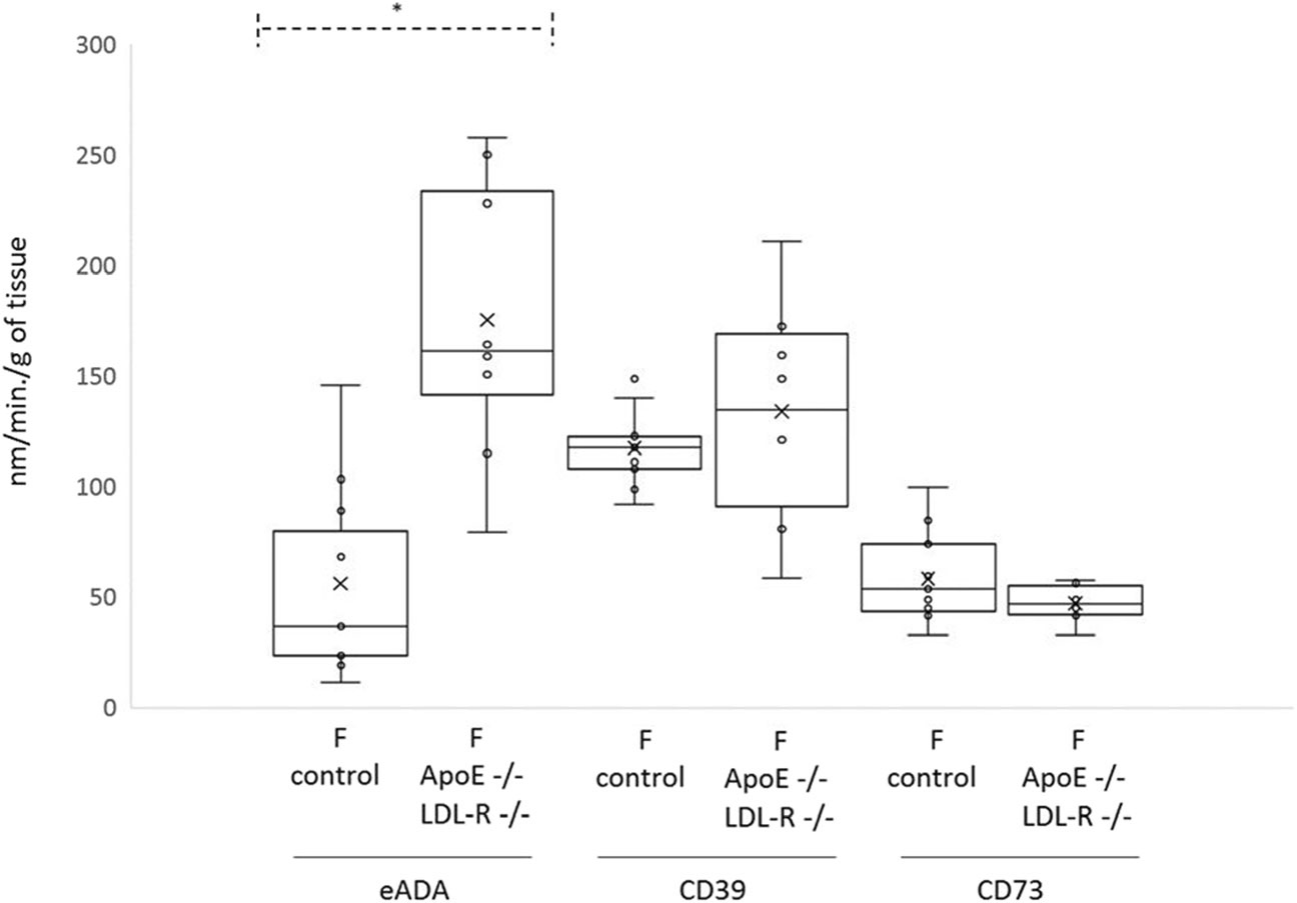
Activities of ADA, CD73, and CD39 in WT (F control) (*n* = 11) and ApoE-/-LDL-R-/- (F LDL-/-ApoE-/-) (*n* = 8) female mice aortic endothelial cells. Data are presented as a boxplots

Extracellular hydrolysis of ATP and AMP catalyzed by CD39 and CD73, respectively, did not differ significantly in aortas of both groups of mice (WT females *n* = 11, female ApoE-/-LDL-R-/- *n*=8) (Table 1, Fig. 3). eADA activity was over three times higher in the ApoE-/-LDL-R-/- group compared with controls 161.3 (79.1–257.7) nm/min/g of tissue in ApoE-/-LDL-R-/- mice vs. 36.6 (11.21–145.7) nm/min/g of tissue in controls, *p* = 0.001 (Table 1, Fig. 2).

## Discussion

The results of the current study show that estrogens may have an impact on extracellular metabolism of purine nucleotides and nucleosides in mice aorta through the regulation of adenosine deaminase activity.

It is known that the estrogens differently influence the function of VSMCs and ECs, depending on sex, and the distribution and expression of estrogen receptors seem to be one of the most important factors. Estradiol increases the production of NO and PGI2 and inhibits the release of TXA2 and superoxide in the endothelium, while in VSMCs, it regulates the function of K^+^ channels [29]. It is known that estrogens may modulate the activity of a vascular purinergic system, although there was no evidence of estrogen participation in the control of the extracellular nucleotide metabolism specifically in the aorta. Thus, we measured activities of three ectoenzymes participating in extracellular metabolism of purine nucleotides and nucleosides in male and female mouse aortas. No differences between males and females in CD39 and CD73 activities were observed, but eADA activity was much higher in males. To our knowledge, this is the first investigation showing the sex difference of eADA activity in mice. Since eADA is responsible for the deamination of adenosine to inosine, it controls the local extracellular adenosine concentration, resulting in a protective effect on the vascular system [5, 10, 30]. Lower eADA activity in females thus would be beneficial, since it improves endothelium function and protects it from the development of inflammatory states. To confirm the influence of estrogens on eADA activity, we incubated female mouse aortas in the presence of estradiol. After 48 h, we observed the diminished adenosine hydrolysis in samples incubated with estradiol compared with those deprived of estradiol. No differences were observed in the activities of CD39 or CD73, which suggests that the production of adenosine is independent of sex. The mechanism of eADA activity regulation by estradiol is unknown, but several possibilities come to mind. It seems that this mechanism is specific for eADA, since CD39 and CD73 activities were not influenced by estradiol. ADA produced intracellularly is released to the plasma membrane to form a complex with CD26 (DPP4), eADA, responsible for the deamination of adenosine to inosine. The latter is different from other ectoenzymes, since CD39 is an integral membrane protein and CD73 is connected to the membrane via a GPI anchor [31, 32]. It is known that in females, the level of endothelial DPP4 is lower than that in men [33]. Furthermore, in male mice, the activity of DPP4 in the microvascular endothelium was much higher than in females [34]. Inhibitors of DPP4 or high levels of estrogens exert a similar protective effect on the vascular endothelium [35]. Based on this information, one may speculate that estradiol could repress DPP4 gene transcription by inhibition of NFκB signaling through the ERα receptor [36, 37], although alternative mechanisms should not be excluded.

With the knowledge that estradiol and adenosine are both involved in cardioprotective processes and that lower levels of both are connected to development of atherosclerosis, we measured activities of all three ectoenzymes in ApoE-/-LDL-R-/- atherogenic female mouse aortas. Both mutations result in the development of atherosclerosis due to hyperlipidemia and also result in disturbances of purinergic signaling [14, 16, 35]. Diminished activity of CD39 and CD73, as observed in thoracic aorta of mice, leads to a lower synthesis of adenosine [14, 16]. However, we saw no differences in activities of CD39 and CD73 in ApoE-/-LDL-R-/- and WT females, and eADA activity was much higher in ApoE-/-LDL-R-/- than that in WT females. The latter results are similar to the effects observed in ApoE-/-LDL-R-/- male mice [35]. Our results derived from measurement of CD39 and CD73 activities are not compatible with those obtained by previous researchers. However, given that in the earlier studies mice of both sexes were pooled, we cannot compare the results [14, 16]. Increased eADA activity was rather unexpected, since the level of endogenous estradiol is within the same range in both groups. We speculate that this may reflect the influence of factors other than estrogens in eADA control in ApoE-/-LDL-R-/- mice, for example, in the appearance of an inflammatory state during development of atherosclerosis, which may influence the interaction of estradiol with the purinergic system [36–38].

The results presented in this paper seem to be a good introduction for further investigations explaining the differences between males and females in the control of cardiovascular system homeostasis and the development of cardiovascular diseases. It also highlights eADA as an important regulatory element in the cardiovascular system. The explanation of gender differences in the control of the purinergic system may be of great importance in the future, e.g., in the design of therapies for cardiovascular diseases dedicated specifically to women.

The critical time for women is menopause, since it is characterized by the decrease of estrogen biosynthesis and concentration in blood and is correlated with increased risk for the development of cardiovascular diseases. Hormone replacement therapy, by increasing the level of circulating estrogen, may help to maintain the proper adenosine level in the blood, thus exerting a protective effect against inflammation and endothelium dysfunction. Additionally, eADA inhibitors, which increase adenosine levels, seem to be useful protective agents. Antibodies against eADA could additionally be an interesting approach.

We acknowledge that the results presented in this paper are only preliminary and that, in the future, we should focus on explaining the mechanisms involved in the influence of estrogens on the purinergic system in mouse (male and female) aortas, since we did not investigate them. One of the limitations of this study is the small sample size. We also did not measure serum estradiol levels of the animals tested, since we assumed that premenopausal females have a naturally higher level of estradiol than males. Because we measured eADA activity in female mouse aortas using 100 nM estrogen concentration only, and we did not study eADA activity in females of different ages (e.g., after menopause), we are not able to discuss the effects of different estradiol concentrations on the aortic purinergic system. However, we plan to investigate that problem in the future. We must note that the results of our study should not be translated directly to humans due to possible discrepancies between human and mice extracellular purine metabolism and in the amount and activity of estrogen receptors.

In conclusion, we demonstrated for the first time that female mice have lower activity of extracellular eADA than males, which is thought to have a beneficial effect. We also showed the probable connection between high estradiol levels and low activity of extracellular eADA. Other ectoenzymes, such as CD39 and CD73, do not exhibit the same regulation pattern. Based on our observation of the differences in eADA activity in WT and female ApoE-/-LDL-R-/- mice, we speculate that factors other than estradiol are involved in the development of atherosclerosis.

## Data Availability

Not applicable.
